# Integrated mechanical and metabolic limb salvage for advanced diabetic Charcot hindfoot collapse with non-traumatic calcaneal fracture: an 8-year follow-up case report

**DOI:** 10.1186/s12891-026-10193-5

**Published:** 2026-07-09

**Authors:** Koji Nozaka, Hiroshi Aonuma, Kenta Tominaga, Naohisa Miyakoshi

**Affiliations:** Department of Orthopaedic Surgery, Akita University Graduate School of Medicine, Akita, Japan

**Keywords:** Charcot neuro-osteoarthropathy, Hindfoot, Calcaneal fracture, Type 1 diabetes mellitus, Circular external fixation, Achilles tendon lengthening, Vitamin D deficiency, Teriparatide, Low-intensity pulsed ultrasound, Limb salvage

## Abstract

**Background:**

Advanced Charcot neuro-osteoarthropathy involving the hindfoot is associated with severe deformity, instability, ulceration, infection, and major lower-limb amputation. Non-traumatic calcaneal fracture associated with diabetic Charcot hindfoot collapse is uncommon and difficult to treat because of neuropathy, impaired bone quality, tendon imbalance, soft tissue compromise, and a high risk of fixation failure. Reports describing long-term functional limb preservation in patients with advanced Charcot hindfoot collapse associated with severe metabolic bone impairment remain limited.

**Case presentation:**

A 63-year-old Japanese woman with a 36-year history of type 1 diabetes mellitus presented with progressive right hindfoot deformity and ulceration without major trauma. Radiographs and computed tomography demonstrated advanced Charcot hindfoot collapse with a non-traumatic calcaneal fracture classified as Eichenholtz stage III and Brodsky type 3B. Preoperative evaluation demonstrated severe vitamin D deficiency and elevated bone turnover markers. The patient underwent corrective osteotomy of the calcaneus, Achilles tendon Z-lengthening, internal fixation with headless compression screws, autologous iliac bone grafting, and circular external fixation. A staged postoperative unloading protocol was used, followed by gradual protected loading. Sequential metabolic therapy was administered after surgery. Bone union was achieved at approximately 3 months, and the external fixator was removed at 3.5 months. During long-term follow-up, glycaemic control and bone mineral density improved. At 8 years after surgery, the patient remained ambulatory with preserved plantigrade alignment and without recurrent ulceration, infection, fixation failure, or amputation. The American Orthopaedic Foot & Ankle Society ankle-hindfoot score was 94. Interpretation of pain scores was limited by profound diabetic neuropathy.

**Conclusions:**

This case illustrates an integrated multidisciplinary limb-salvage strategy combining mechanical reconstruction, staged postoperative offloading, and metabolic optimisation in a patient with advanced diabetic Charcot hindfoot deformity complicated by severe metabolic bone impairment. The favourable long-term outcome should be interpreted as hypothesis-generating rather than evidence for the efficacy of any individual adjunctive therapy.

## Background

Charcot neuro-osteoarthropathy is a severe complication of diabetic neuropathy characterised by progressive bone and joint destruction, deformity, instability, ulceration, infection, and possible limb loss [1]. Although nonoperative treatment with immobilisation and offloading remains important, advanced deformity involving the ankle and hindfoot is particularly challenging because loss of alignment may compromise plantigrade loading and increase the risk of limb-threatening complications [2, 3].

Delayed recognition of Charcot neuro-osteoarthropathy may contribute to progression of deformity and delayed initiation of appropriate offloading or reconstruction [4]. Once ulceration or infection occurs, management becomes more complex, and careful assessment for diabetic foot infection is essential [5]. In patients with diabetes, ankle and hindfoot fractures are also associated with increased risks of delayed union, nonunion, Charcot progression, wound complications, infection, and amputation [6, 7]. Therefore, maintaining alignment, avoiding recurrent ulceration, and achieving stable union are important goals in diabetic Charcot-associated hindfoot reconstruction.

Non-traumatic calcaneal fracture associated with advanced diabetic Charcot hindfoot collapse is relatively uncommon and difficult to reconstruct because of severe neuropathy, abnormal loading mechanics, impaired bone quality, and soft tissue compromise. Achilles tendon contracture may also contribute to progressive displacement of the posterior calcaneal fragment and worsening deformity.

Circular external fixation has been used for complex diabetic foot and ankle reconstruction because it allows stable fixation while limiting additional soft tissue dissection [8, 9]. Arthrodesis remains an important salvage option for severe ankle and hindfoot Charcot deformity, particularly when joint destruction or instability is non-reconstructable [10–12]. However, in selected patients in whom infection is absent, residual joint architecture is preserved, and alignment is reconstructable, joint-preserving reconstruction to restore a plantigrade foot may be considered.

Major lower-limb amputation is associated with substantial mortality and functional decline, particularly in medically fragile patients [13, 14]. Therefore, durable limb preservation, when safely achievable, may have clinical value beyond radiographic union alone. Biological and metabolic adjuncts, including teriparatide and low-intensity pulsed ultrasound, have been discussed as potential options in selected patients with impaired bone healing potential [15, 16], although their independent effects cannot be determined from single-case observations.

Reports describing long-term functional limb preservation beyond 5 years in patients with advanced Charcot hindfoot collapse associated with a non-traumatic calcaneal fracture and severe metabolic bone impairment remain limited. We report a rare case treated using integrated mechanical reconstruction, biological stimulation, and metabolic optimisation, including Achilles tendon balancing, corrective osteotomy, circular external fixation, low-intensity pulsed ultrasound, and sequential osteoporosis therapy, with functional limb preservation maintained at 8 years. The novelty of this report lies not in any individual intervention but in the combination of this unusual pathology, severe metabolic bone impairment, an integrated reconstructive strategy, and the durability of the long-term outcome.

## Case presentation

A 63-year-old Japanese woman with a 36-year history of type 1 diabetes mellitus presented with progressive right hindfoot deformity and ulceration without major trauma. Before fracture onset, she had been independently ambulatory and routinely walked approximately 30 min daily.

Her diabetic history was complicated by advanced microvascular and neurological complications, including diabetic retinopathy requiring surgery for vitreous haemorrhage, profound peripheral neuropathy, and autonomic dysfunction with orthostatic hypotension. She had no smoking history and consumed alcohol only occasionally. Her body height was 150 cm, body weight was 43 kg, and body mass index was 19.1 kg/m².

**Fig. 1:**
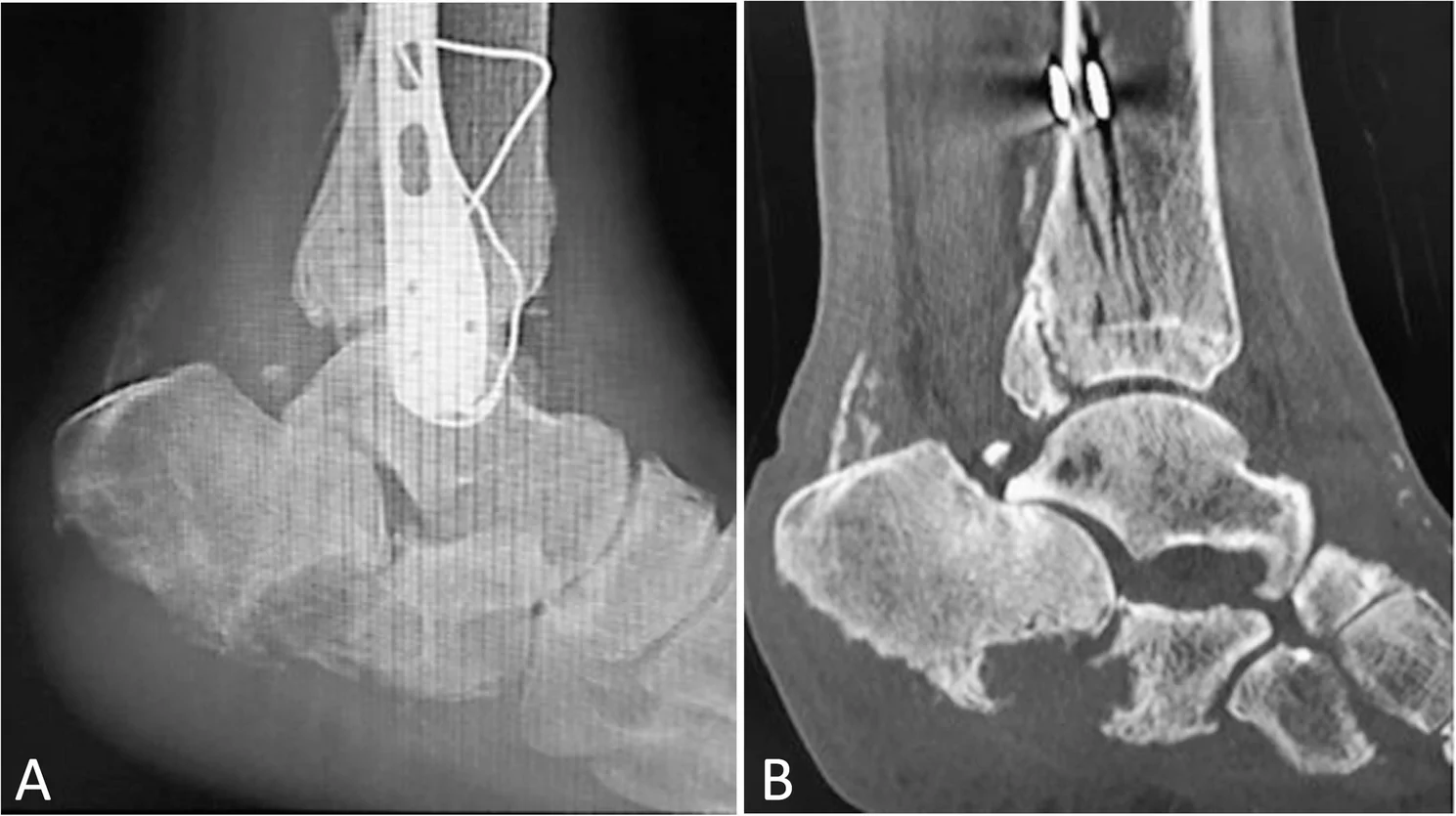
Initial imaging findings obtained at the referring hospital. **A** Lateral radiograph demonstrating a markedly displaced fracture through the body of the calcaneus. **B** Sagittal computed tomography image demonstrating severe displacement of the posterior calcaneal fragment associated with advanced Charcot hindfoot collapse

**Fig. 2:**
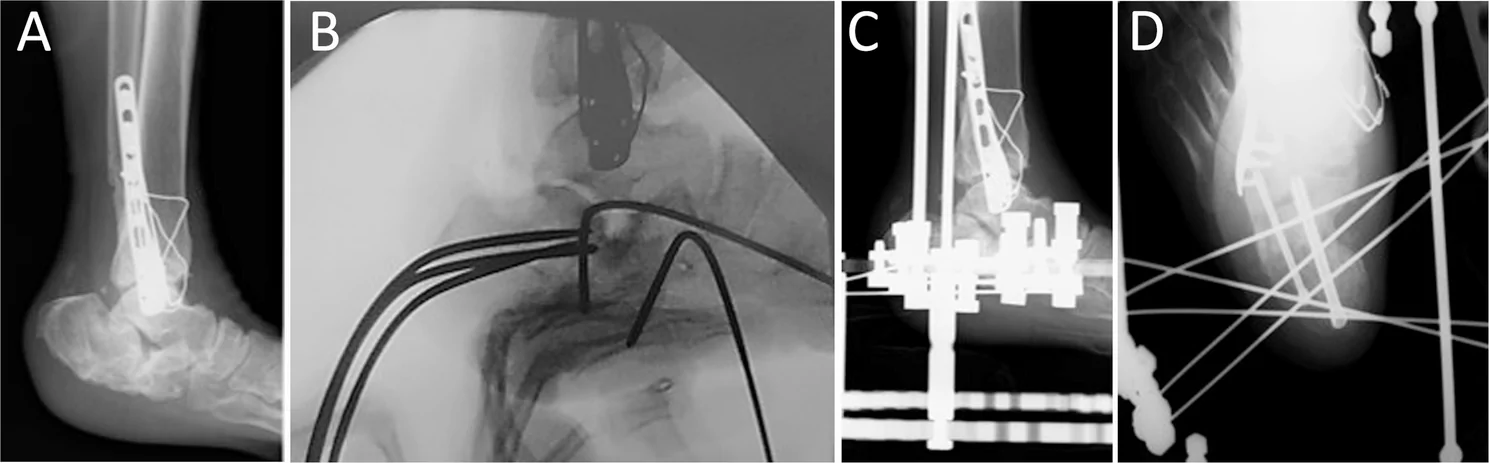
Surgical reconstruction after referral to our institution. **A** Lateral radiograph obtained at the first visit to our institution, 6 months after the radiograph shown in Fig. 1, demonstrating calcaneal malunion and deformity. **B** Intraoperative fluoroscopic image showing corrective osteotomy performed at the calcaneal fracture malunion site. After Achilles tendon lengthening, correction was achieved using the joystick technique with three 3.0-mm Kirschner wires. **C**, **D** The calcaneus was stabilised using two headless compression screws and additional fixation with five tensioned wires connected to the foot ring

Despite the absence of subjective numbness, clinical examination demonstrated markedly impaired protective sensation. Vibration sense was 8 s on the affected side and 9 s on the contralateral side. Formal 10-g monofilament testing was not documented. Dorsalis pedis pulses were palpable bilaterally. Ulceration associated with hindfoot deformity was documented clinically at presentation, although baseline ulcer photographs were not archived. Initial radiographs and computed tomography obtained at the referring hospital demonstrated a markedly displaced fracture through the body of the calcaneus and displacement of the posterior calcaneal fragment (Fig. 1). At the first visit to our institution 6 months later, lateral radiographs demonstrated calcaneal malunion and deformity (Fig. 2A). The deformity was classified as Eichenholtz stage III and Brodsky type 3B.

The patient had chronically suboptimal glycaemic control, with a haemoglobin A1c level of 8.0% despite ongoing insulin therapy. Because of concern regarding perioperative glycaemic control, she underwent preoperative hospitalisation under diabetologist supervision approximately 1 month before surgery. Sliding-scale insulin therapy using insulin aspart was administered three times daily for perioperative glycaemic optimisation. A detailed timeline of the clinical course and interventions is shown in Table 1.

**Table 1:** Timeline of interventions and follow-up

Time point	Intervention or assessment	Key findings or management details
Initial assessment at referring hospital	Radiographs and CT	Markedly displaced non-traumatic calcaneal fracture with posterior fragment displacement.
Approximately 6 months later	Referral to our institution	Calcaneal malunion and advanced Charcot hindfoot collapse; Eichenholtz stage III and Brodsky type 3B.
Preoperative period	Diabetology admission and metabolic assessment	HbA1c 8.0%; 25-OHD 7 ng/mL; eGFR 50.8 mL/min/1.73 m²; no laboratory evidence of active infection.
Approximately 1 month before surgery	Glycaemic optimisation	Sliding-scale insulin therapy using insulin aspart three times daily under diabetologist supervision.
Index surgery	Reconstruction	Calcaneal corrective osteotomy, Achilles tendon Z-lengthening, headless screw fixation, iliac bone grafting, and circular external fixation.
Postoperative day 0 to 4 weeks	Unloading strategy	Double foot rings used to avoid plantar calcaneal weight-bearing; ambulation without plantar loading permitted.
From 4 weeks postoperatively	Staged loading	Plantar-contact walking initiated using a plantar orthosis while maintaining external fixation support.
Immediately after surgery to 1 year	Biological adjunct	Low-intensity pulsed ultrasound applied for 20 min daily.
Approximately 3 months postoperatively	Bone union	Radiographic bone union observed.
3.5 months postoperatively	Frame removal	External fixator removed; ankle-foot orthosis applied for an additional 4 weeks.
Postoperative metabolic therapy	Sequential osteoporosis therapy	Teriparatide with active vitamin D for 1.5 years, followed by annual zoledronic acid therapy after improvement of glycaemic control and renal function.
8-year follow-up	Final assessment	Plantigrade alignment maintained; no recurrent ulceration, infection, fixation failure, or amputation; AOFAS ankle-hindfoot score 94.

Preoperative laboratory examination demonstrated no evidence of active infection, with a normal white blood cell count and C-reactive protein level (< 0.03 mg/dL). Severe vitamin D deficiency was identified, with a serum 25-hydroxyvitamin D level of 7 ng/mL. Bone turnover markers suggested high-turnover bone metabolism, including elevated tartrate-resistant acid phosphatase-5b (841 mU/dL) and total procollagen type I N-terminal propeptide (111 µg/L). Serum intact parathyroid hormone level was 34 pg/mL. Renal function was mildly impaired, with an estimated glomerular filtration rate of 50.8 mL/min/1.73 m². Key metabolic and laboratory data are summarised in Table 2.

**Table 2:** Key laboratory, metabolic, and bone mineral density data

Parameter	Preoperative / before treatment	Latest follow-up / long-term course	Comment
HbA1c (%)	8.0	6.1	Improved during long-term follow-up.
25-hydroxyvitamin D (ng/mL)	7	Not available	Repeated testing was not routinely reimbursed within the Japanese healthcare system during the treatment period.
Intact PTH (pg/mL)	34	40	Remained within the reference range.
TRACP-5b (mU/dL)	841	352	Elevated preoperatively, suggesting high bone turnover.
Total P1NP (µg/L)	111	82	Elevated preoperatively, suggesting high bone turnover.
eGFR (mL/min/1.73 m²)	50.8	70.4	Zoledronic acid was administered after improvement of renal function, with monitoring.
Femoral neck BMD (g/cm²)	0.563	0.604	Improved during sequential osteoporosis therapy.
Femoral neck T-score	-2.1	-1.7	Improved during sequential osteoporosis therapy.
CRP (mg/dL)	< 0.03	No recurrence of infection	No laboratory evidence of active infection preoperatively.

Surgical reconstruction was performed using an extensile lateral approach to the subtalar region. After fluoroscopic identification of the deformity apex, multiple drill holes were created and osteotomy was completed using an osteotome. Three 3.0-mm Kirschner wires were inserted into the posterior calcaneal fragment, and gradual correction was performed using the joystick technique. Residual Achilles tendon tension limited reduction; therefore, distal Z-lengthening of the Achilles tendon was additionally performed. The tendon was repaired using FiberWire with the Krackow suture technique (Fig. 2B).

Following improvement of alignment, definitive fixation was achieved using one 6.5-mm and one 4.5-mm headless compression screw inserted from posteriorly. Autologous iliac crest bone grafting was performed at the osteotomy site. Because isolated internal fixation alone was considered potentially insufficient in the setting of advanced Charcot instability and diabetic bone fragility, circular external fixation was additionally applied. Five tensioned wires were used to stabilise the calcaneus to the foot ring (Fig. 2C, D).

**Fig. 3:**
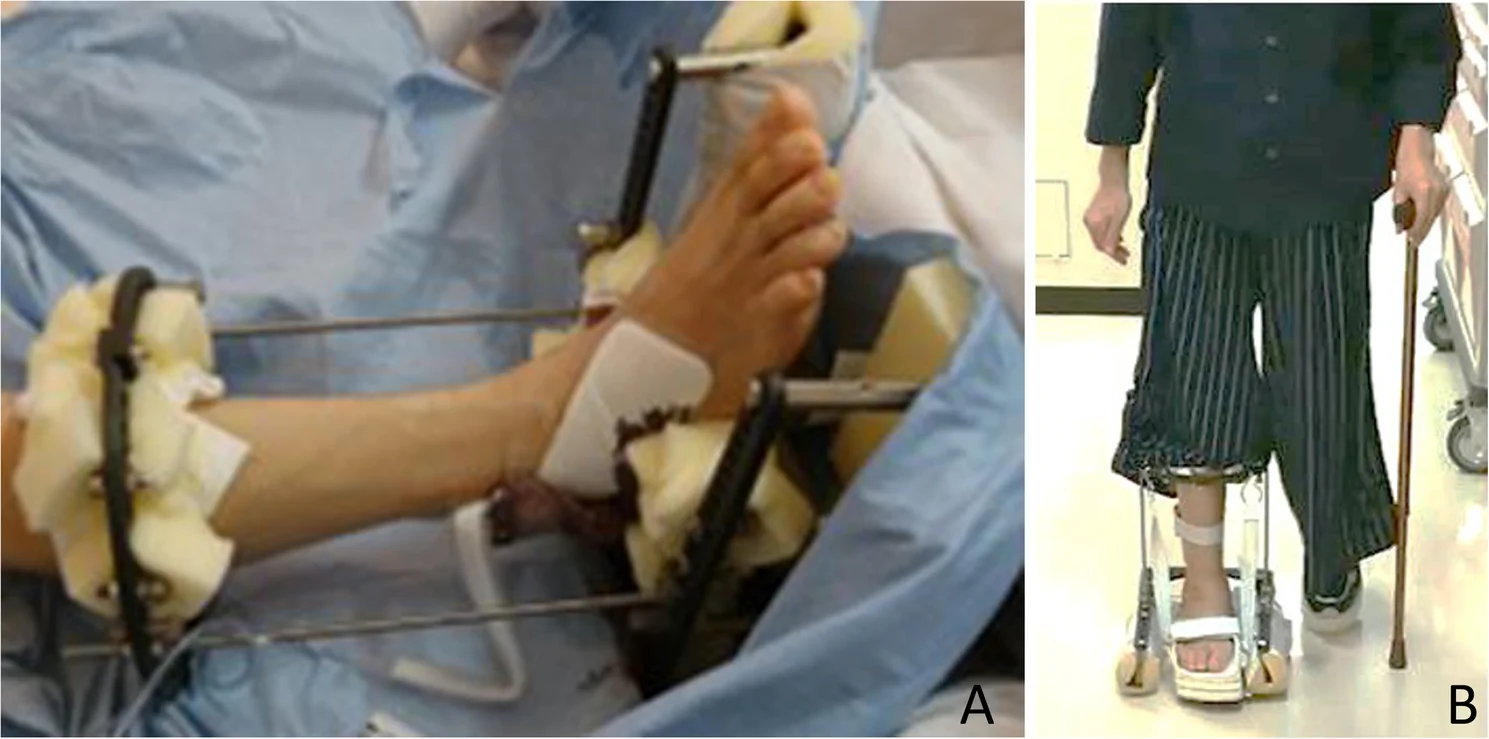
Postoperative management and staged loading strategy. **A** Immediate postoperative clinical photograph demonstrating the double foot-ring construct. Plantar contact was avoided during the first 4 postoperative weeks, and ambulation without plantar loading was permitted. Incisional negative pressure wound therapy was applied to the surgical wound. **B** Clinical photograph obtained 4 weeks postoperatively. Plantar-contact walking was initiated using a plantar orthosis while maintaining external fixation support

Double foot rings were used during the first 4 postoperative weeks to maintain complete hindfoot unloading and avoid plantar calcaneal weight-bearing. Ambulation without plantar loading was permitted during this period. Incisional negative pressure wound therapy was applied to the surgical wound (Fig. 3A). From 4 weeks postoperatively, plantar-contact walking was initiated using a plantar orthosis while maintaining external fixation support (Fig. 3B).

Low-intensity pulsed ultrasound (LIPUS) was initiated immediately after surgery as an adjunctive biological stimulation therapy. A SAFHS 2000 device (Exogen, Inc., Piscataway, NJ, USA) was used for 20 min daily. The ultrasound parameters were a frequency of 1.5 MHz, a signal burst width of 200 microseconds, a signal repetition frequency of 1 kHz, and an intensity of 30 mW/cm². The transducer was applied directly over the reconstructed calcaneal fracture site, and treatment was continued daily for 1 year. Bone union was observed at approximately 3 months postoperatively, and the external fixator was removed at 3.5 months. An ankle-foot orthosis was applied for an additional 4 weeks after frame removal.

**Fig. 4:**
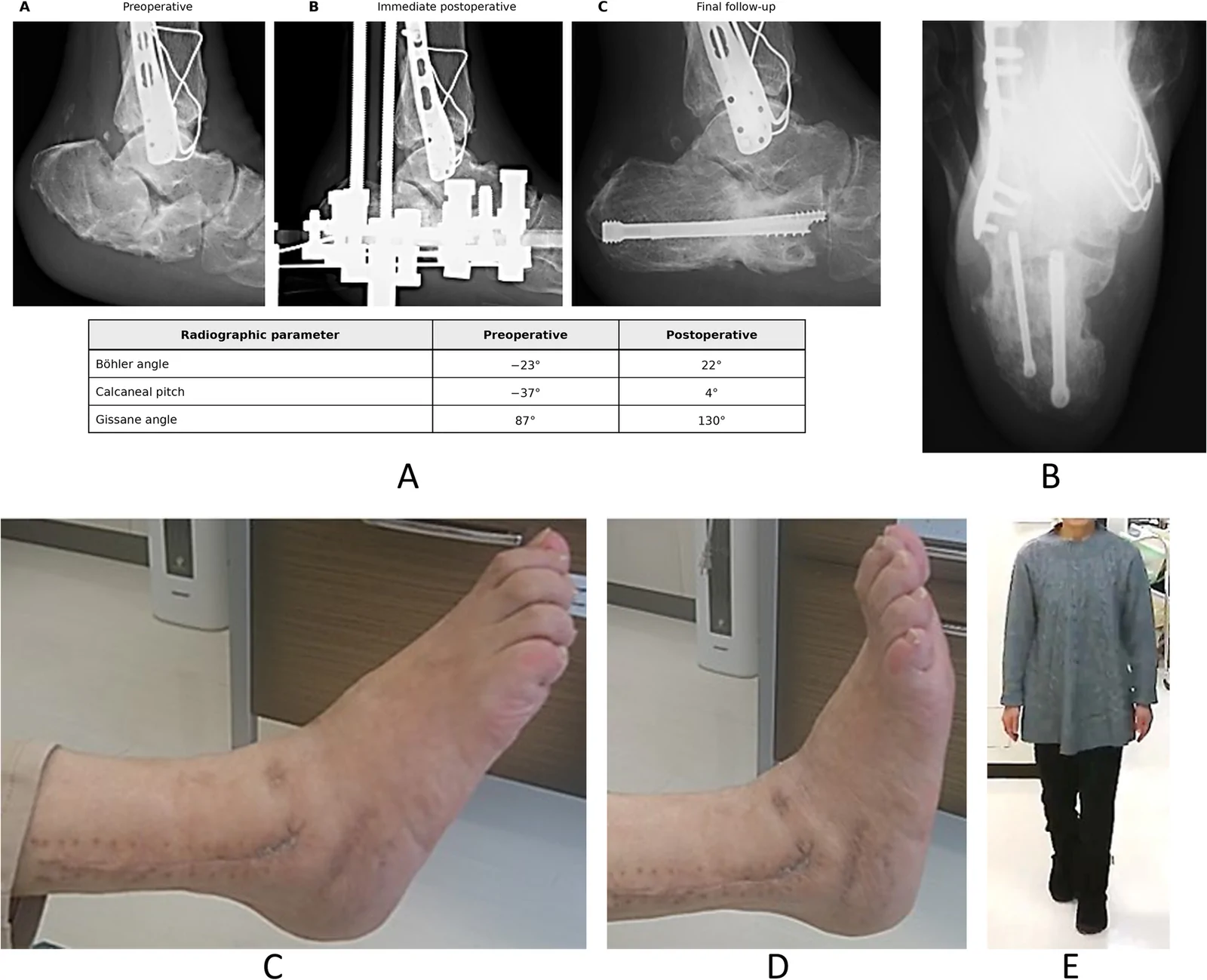
Radiographic and clinical outcomes after reconstruction. **A** Comparative lateral radiographs demonstrating severe preoperative calcaneal collapse, restoration of alignment immediately after reconstruction, and maintenance of correction at the final follow-up. Quantitative radiographic parameters including Böhler angle, calcaneal pitch, and Gissane angle are shown. **B** Axial radiograph obtained at the final follow-up demonstrating maintenance of hindfoot alignment. **C**, **D** Final clinical photographs demonstrating preserved ankle plantarflexion and dorsiflexion. **E** Clinical photograph demonstrating independent ambulation without use of a cane

Radiographic parameters showed substantial improvement following reconstruction. Böhler angle improved from − 23° preoperatively to 22° postoperatively, calcaneal pitch improved from − 37° to 4°, and Gissane angle increased from 87° to 130° (Fig. 4A).

Longitudinal bone mineral density assessment demonstrated gradual improvement during sequential osteoporosis therapy. Femoral neck bone mineral density increased from 0.563 g/cm² before treatment to 0.604 g/cm² at the latest follow-up, while the T-score improved from − 2.1 to -1.7. Glycaemic control also improved during follow-up, with haemoglobin A1c decreasing from 8.0% preoperatively to 6.1%. Teriparatide combined with active vitamin D supplementation was administered for 1.5 years under careful monitoring of renal function and serum calcium levels, followed by annual zoledronic acid infusion therapy after improvement of glycaemic control and renal function.

At the final follow-up 8 years after surgery, the patient remained ambulatory with preserved plantigrade alignment and no recurrence of ulceration, infection, fixation failure, or amputation. Radiographs demonstrated solid bone union without evidence of adjacent joint degeneration (Fig. 4A, B). Clinical photographs demonstrated preserved ankle plantarflexion and dorsiflexion, and the patient was able to walk independently without a cane (Fig. 4C-E). The American Orthopaedic Foot & Ankle Society ankle-hindfoot score was 94, and the visual analogue scale pain score was 0/100. However, interpretation of pain severity was limited because severe neuropathy had resulted in minimal pain even before surgery. Patient-reported quality-of-life assessment using the Short Form-36 questionnaire demonstrated relatively preserved role functioning, social participation, emotional status, and mental health despite residual physical limitations.

## Discussion

Advanced diabetic Charcot hindfoot collapse remains one of the most challenging limb salvage conditions in orthopaedic surgery because of severe neuropathy, impaired bone quality, abnormal loading mechanics, soft tissue compromise, ulceration, infection risk, and possible major amputation [1–7]. Hindfoot involvement may be particularly problematic because collapse of the subtalar and ankle complex can lead to profound instability, abnormal plantar pressure, recurrent ulceration, infection, and eventual limb loss [2].

The present case had several high-risk features, including longstanding type 1 diabetes mellitus, severe neuropathy, ulceration, autonomic dysfunction, advanced hindfoot deformity, and profound vitamin D deficiency. Nevertheless, prolonged limb preservation was maintained for 8 years following multidisciplinary management. This case may be clinically relevant because it illustrates a staged reconstruction strategy that addressed mechanical deformity, soft tissue protection, postoperative unloading, and metabolic bone fragility in a patient at high risk for recurrent collapse or fixation failure.

Another clinically important aspect of this case was the delayed recognition of the calcaneal fracture. At presentation to our institution, approximately 6 months had elapsed since the initial radiographic assessment, and progressive calcaneal malunion and hindfoot collapse had already developed. Profound sensory neuropathy may have contributed to delayed presentation because substantial structural collapse occurred despite minimal pain.

A particularly important mechanical feature in this case was Achilles tendon imbalance. Reduction improved after Achilles tendon lengthening, suggesting that Achilles-driven deforming forces may have influenced hindfoot instability and posterior fragment displacement. Careful correction using Kirschner wires and staged reduction may also have minimised additional osseous injury in fragile Charcot bone. However, because this is a single case, the relative mechanical contribution of Achilles tendon lengthening to long-term maintenance of alignment cannot be determined.

Circular external fixation was selected because isolated internal fixation alone was considered potentially insufficient in the setting of diabetic Charcot bone fragility. The circular external fixation construct provided additional mechanical protection while minimising further soft tissue compromise. Previous reports have suggested that the use of circular external fixation may be useful in diabetic ankle and Charcot-related reconstruction, particularly when soft tissue condition or vascularity raises concern regarding extensive open surgery [8, 9].

Arthrodesis is an established option for severe ankle and hindfoot pathology and may provide reliable stability in non-reconstructable deformity [10–12]. However, fusion or primary amputation may not be the only option in selected patients when infection is absent, alignment can be restored, and a plantigrade foot can be maintained. In the present case, joint-preserving reconstruction was considered feasible because active infection was absent and the principal pathology was located within the calcaneus. Although severe hindfoot deformity was present, advanced destruction of the subtalar or ankle joints was not observed, and restoration of a plantigrade foot was considered achievable through corrective osteotomy and protected fixation. Therefore, reconstruction of the calcaneal deformity was preferred over primary hindfoot or tibiotalocalcaneal arthrodesis.

In comparison with previous reports describing circular fixation or arthrodesis for Charcot-related ankle and hindfoot pathology [8–12], the present case differs in that the main pathology was a non-traumatic calcaneal fracture and malunion with residual joint architecture rather than complete ankle or subtalar joint destruction. The educational value of this case therefore lies in the decision-making process for selected joint-preserving reconstruction, the staged postoperative unloading strategy, and the integration of metabolic optimisation in a high-risk diabetic patient.

Furthermore, the staged postoperative unloading protocol using double foot rings enabled temporary hindfoot suspension during the first 4 postoperative weeks. Thereafter, plantar-contact walking was initiated with a plantar orthosis while maintaining external fixation support, allowing gradual transition from complete unloading to protected loading. This staged protocol was selected because immediate plantar loading on the reconstructed calcaneus was considered potentially risky owing to calcaneal involvement, ulceration, and severe metabolic bone impairment.

Another notable feature of this case was severe metabolic bone impairment. Profound vitamin D deficiency and elevated bone turnover markers suggested metabolically active diabetic bone fragility that may have contributed to progressive Charcot collapse. Mechanical reconstruction alone may therefore have been insufficient to maintain long-term structural stability.

In addition to mechanical stabilisation, biological and metabolic support was incorporated. Teriparatide has been reported as a potential treatment option for delayed union and nonunion [15], and low-intensity pulsed ultrasound has also been investigated as an adjunct for fracture healing [16]. In the present case, low-intensity pulsed ultrasound was initiated immediately after surgery and continued for 1 year. Sequential metabolic therapy consisting of teriparatide, active vitamin D supplementation, and subsequent annual zoledronic acid administration was also introduced in an attempt to address severe metabolic bone fragility.

Metabolic therapy was administered with careful monitoring of renal function and serum calcium levels because the patient initially demonstrated impaired renal function and severe metabolic bone fragility. The independent contribution of low-intensity pulsed ultrasound, teriparatide, active vitamin D, and zoledronic acid cannot be determined from a single case, and the favourable clinical course should not be interpreted as evidence for the efficacy of any individual adjunctive therapy. Rather, these treatments formed part of a broader multidisciplinary attempt to optimise the local mechanical and systemic metabolic environment. Serial bone mineral density measurements demonstrated gradual improvement during long-term metabolic therapy, with femoral neck bone mineral density increasing from 0.563 g/cm² to 0.604 g/cm² and T-score improving from − 2.1 to -1.7. Glycaemic control also improved substantially during follow-up, with haemoglobin A1c decreasing from 8.0% to 6.1%. Although causal relationships between pharmacological treatment and clinical outcome cannot be established from a single case, these findings suggest successful long-term metabolic optimisation.

The favourable long-term course observed in this case suggests that an integrated multidisciplinary approach may be considered in selected patients with advanced diabetic Charcot hindfoot deformity, although the contribution of individual treatment components remains uncertain. The findings should therefore be interpreted as hypothesis-generating rather than evidence of efficacy for any individual intervention.

Avoidance of major amputation may also be clinically meaningful in medically fragile patients. Previous studies have reported high short- and long-term mortality after lower-limb amputation and substantial functional decline after amputation [13, 14]. Although amputation may be necessary in cases with uncontrolled infection, non-reconstructable deformity, or non-salvageable soft tissue conditions, long-term ambulatory limb preservation may be valuable when it can be achieved safely.

Another notable aspect of the present case was the favourable long-term functional outcome. At 8 years postoperatively, the patient maintained ambulatory ability with an American Orthopaedic Foot & Ankle Society ankle-hindfoot score of 94. Although the visual analogue scale pain score was 0/100, pain interpretation should be approached cautiously because profound diabetic neuropathy may mask symptoms in Charcot neuro-osteoarthropathy. Furthermore, pain was minimal even before reconstruction despite advanced structural deformity, indicating that postoperative pain scores may underestimate disease severity and should not be interpreted as an isolated measure of treatment success. Nevertheless, the Short Form-36 profile suggested relatively preserved role functioning, social participation, emotional status, and mental health despite residual physical limitations. These findings may indicate that long-term limb preservation contributed not only to structural maintenance but also to preservation of independence and quality of life.

Successful long-term limb preservation in Charcot reconstruction may require not only correction of deformity and stabilisation, but also multidisciplinary perioperative optimisation addressing diabetic metabolic fragility, soft tissue protection, biological support, functional rehabilitation, and postoperative offloading. The treatment strategy described in this report does not represent a formal institutional protocol but reflects the authors’ reconstructive principles for selected patients with complex Charcot hindfoot deformity, including restoration of alignment, protection of soft tissues, staged postoperative offloading, and optimisation of metabolic bone health.

## Limitations

This report has several limitations. First, this is a single-case report, and the relative contributions of surgical reconstruction, Achilles tendon lengthening, postoperative offloading, glycaemic optimisation, low-intensity pulsed ultrasound, and sequential metabolic therapy cannot be determined independently. Second, although serial bone mineral density measurements were available, detailed assessment of the independent effects of each metabolic intervention was not possible. Third, because no control comparison exists, the generalisability of this treatment strategy remains uncertain. Fourth, baseline ulcer photographs were unavailable because no clinical images had been archived at the time of presentation. Fifth, formal 10-g monofilament testing and other quantitative sensory testing data were not available. Sixth, serial 25-hydroxyvitamin D measurements were unavailable because repeated testing was not routinely reimbursed within the Japanese healthcare system during the treatment period. Seventh, interpretation of pain-related outcomes should be performed cautiously because profound diabetic neuropathy may have masked symptoms before and after reconstruction. Eighth, although the Short Form-36 questionnaire provided useful patient-reported information, formal norm-based scoring was not performed in this report. Ninth, although the American Orthopaedic Foot & Ankle Society ankle-hindfoot score remains widely reported in foot and ankle literature, it includes physician-derived components and may not fully capture the patient’s subjective perception of function and quality of life. Nevertheless, the prolonged 8-year limb preservation without recurrent ulceration or amputation, together with favourable functional and patient-reported quality-of-life findings, may provide clinically relevant information regarding multidisciplinary management of advanced Charcot hindfoot deformity.

## Conclusions

We describe a rare case of advanced diabetic Charcot hindfoot collapse with non-traumatic calcaneal fracture treated using integrated mechanical reconstruction, biological stimulation, and metabolic optimisation. Long-term functional limb preservation over 8 years was observed following corrective osteotomy, Achilles tendon balancing, circular external fixation, staged postoperative offloading, low-intensity pulsed ultrasound, and sequential osteoporosis therapy. Although causal relationships cannot be established from a single case, this multidisciplinary strategy may represent a potential treatment option for selected patients with complex Charcot hindfoot deformity associated with severe metabolic bone fragility. The findings should be interpreted as hypothesis-generating and not as evidence for the efficacy of any individual adjunctive treatment component.

## Data Availability

All data generated or analysed during this study are included in this published article.

## Abbreviations

AOFAS: American Orthopaedic Foot & Ankle Society
CT: Computed tomography
LIPUS: Low-intensity pulsed ultrasound
PTH: Parathyroid hormone
SF-36: Short Form-36
25-OHD: 25-hydroxyvitamin D
VAS: Visual analogue scale
